# Optical Monitoring of *In Situ* Iron Loading into Single, Native Ferritin Proteins

**DOI:** 10.1021/acs.nanolett.3c00042

**Published:** 2023-04-13

**Authors:** Arman Yousefi, Cuifeng Ying, Christopher D. J. Parmenter, Mahya Assadipapari, Gabriel Sanderson, Ze Zheng, Lei Xu, Saaman Zargarbashi, Graham J. Hickman, Richard B. Cousins, Christopher J. Mellor, Michael Mayer, Mohsen Rahmani

**Affiliations:** † Advanced Optics and Photonics Laboratory, Department of Engineering, School of Science and Technology, Nottingham Trent University, Nottingham NG118 NS, United Kingdom; ‡ Nanoscale and Microscale Research Centre, University of Nottingham, Nottingham NG7 2RD, United Kingdom; § School of Science and Technology, Nottingham Trent University, Nottingham NG11 8NS, United Kingdom; ∥ School of Physics and Astronomy, University of Nottingham, Nottingham NG7 2RD, United Kingdom; ⊥ Adolphe Merkle Institute, University of Fribourg, Chemin des Verdiers 4, CH-1700 Fribourg, Switzerland

**Keywords:** ferritin, single-protein interrogation, structural dynamics, optical nanotweezers

## Abstract

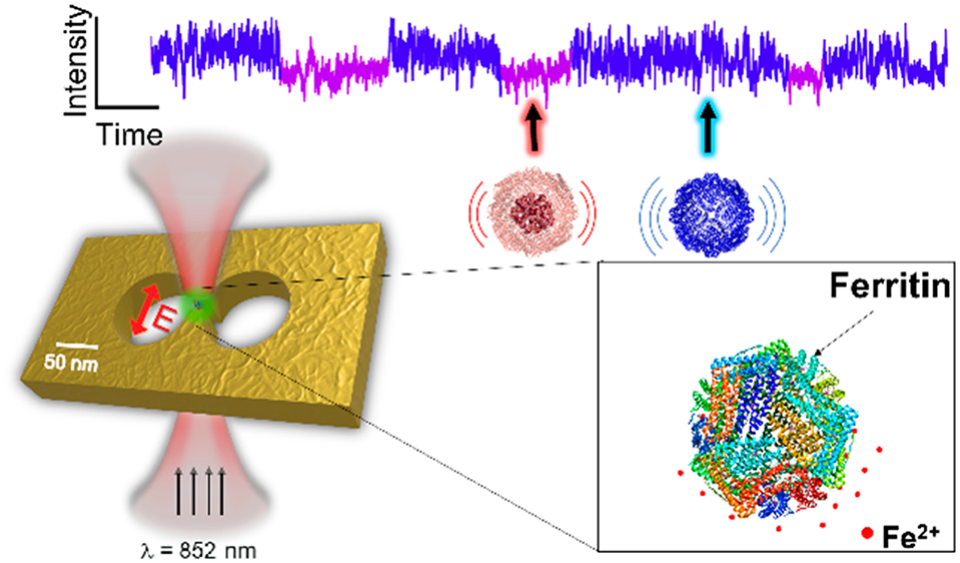

Ferritin is a protein that stores and releases iron to prevent diseases associated with iron dysregulation in plants, animals, and bacteria. The conversion between iron-loaded holo-ferritin and empty apo-ferritin is an important process for iron regulation. To date, studies of ferritin have used either ensemble measurements to quantify the characteristics of a large number of proteins or single-molecule approaches to interrogate labeled or modified proteins. Here we demonstrate the first real-time study of the dynamics of iron ion loading and biomineralization within a single, unlabeled ferritin protein. Using optical nanotweezers, we trapped single apo- and holo-ferritins indefinitely, distinguished one from the other, and monitored their structural dynamics in real time. The study presented here deepens the understanding of the iron uptake mechanism of ferritin proteins, which may lead to new therapeutics for iron-related diseases.

Ferritin is an iron-storage protein that exists in large quantities in bacteria, plants, and the blood of many mammals, including humans.^1−3^ This intracellular protein naturally stores iron and releases it in a controlled fashion. Ferritin plays a key role in preventing diseases and in the detoxification of metals in living organisms.^1^ In humans, for example, iron deficiency can lead to disease states such as anemia, while excess iron may cause increased oxidative stress within cells which can lead to neurodegenerative diseases such as Alzheimer’s disease (AD).^2^ Upon loading iron into its core, ferritin undergoes conformational changes from its apo, iron-free form to its holo, iron-containing form.^3,4^ Therefore, the conversion between apo and holo forms allows ferritin to serve as an iron buffer in living cells.^5,6^ However, the dynamics of such a conversion have not been fully decoded and understood because, to date, no single-molecule techniques are available to detect the globular conformational dynamics of single ferritins without modification.

Today, the experimental evidence of iron accumulation in ferritin cages is mostly obtained by ensemble measurements such as nuclear magnetic resonance (NMR) spectroscopy,^7^ X-ray diffraction,^8^ circular dichroism,^9^ and infrared spectroscopy.^10^ These techniques only enable quantitative study on a large number of proteins. Despite the valuable information about ferritin disclosed by such ensemble techniques,^8,11,12^ they are limited to determining information about the dynamic structural changes during iron loading into the ferritin cage, and the interactions between iron and ferritin.^8,11,12^ Therefore, single-molecule characterization techniques have attracted significant attention due to their capability to provide better insight into molecular mechanisms, a dynamic view of the stochastic nature of chemical processes, and an overview of the heterogeneity across molecular systems.^13−15^ Electron microscopy (EM) techniques such as cryo-scanning transmission EM (Cryo-STEM) have been utilized to reveal the crystallization procedure of ferritin.^16^ This approach takes snapshots of different conformations of many individual ferritin proteins and then reconstructs the dynamic pathway. However, the necessity to run EM in a vacuum prevents this technique from detecting proteins in their native liquid environment. By using graphene liquid cell-transmission electron microscopy (GLC-TEM), Narayanan et al. have demonstrated the biomineralization processes within individual ferritin proteins.^17^ This technique, however, necessitates a further process of ferritin encapsulation between graphene sheets to provide a liquid environment that can impact the dynamics of the protein and induce damage to the protein’s structure.^17,18^ On the other hand, by using conductive probe atomic force microscopy (CP-AFM), Axford et al. demonstrated that holo-ferritin has higher conductivity than apo-ferritin, due to the presence of a metal core inside the holo form.^19^ The main limitation of this approach is the high stiffness of the AFM cantilever, which limits it from studying small domain movements or conformational changes in proteins.^20^

Despite the invasive nature of the aforementioned techniques for single-molecule characterization, they have revealed important information about ferritin. Ferritin is a spherical protein composed of 24 identical subunits with an outer diameter of ∼12 nm and an inner diameter of ∼8 nm.^21^ The self-assembly of these subunits results in the formation of two important types of channels, namely, 3-fold and 4-fold channels, which serve as pathways for ions or molecules to enter and exit the inner core of ferritin proteins.^22^ The 3-fold channels facilitate ion transport and house the site of ferroxidase activity, which occurs through the oxidation of Fe^2+^ to Fe^3+^ via O_2_ or H_2_O_2_.^21^ Additionally, the acidic residues within the ferritin cavity create a negatively charged interface that enables the incorporation of up to 4500 Fe^3+^ ions and the formation of inorganic crystals.^21,22^

With the development of molecular dynamics (MD) simulation and theoretical prediction, there has been more important information about ferritin revealed, particularly the dynamics of the accumulation process of metal ions as well as conformational changes in the amino acid side chains.^23^ For example, the mechanism of metal-binding and biomineralization in the ferritin cage has recently been investigated by theoretical calculations and simulations.^24,25^ Molecular dynamic studies, however, are typically limited by short time scales (i.e., ∼nanoseconds). To the best of our knowledge, there has been no experimental demonstration of structural dynamics following the conversion between apo- and holo-ferritins at a single-molecule level.

The work presented here provides the first experimental observation of the difference in dynamics between individual, unlabeled apo- and holo-ferritins in a liquid environment. We employed a homemade characterization setup, plasmonic nanotweezers using a double-nanohole (DNH) nanostructure.^26−29^ The setup enables confining the optical field into nanometer hot spots with the DNH, generating a large electric field gradient to hold single proteins without diffusing away. In addition, the nanoaperture approach provides label-free detection, allowing monitoring of the dynamics of the trapped protein in a physiological solution.^29^ We demonstrate that holo-ferritin possesses a more rigid, compact conformation compared to its apo counterpart.

Importantly, this work offers the first dynamic tracking of the iron mineralization of single ferritins in real time without any modification of the proteins. The iron regulation or nanotechnology-related applications of ferritin mostly rely on the controllable loading and release of iron ions (or other targeted ions and biomolecules).^30^ In addition, the hollow shell structure of apo-ferritin makes it an ideal template for drug delivery and bioimaging in cancer cells.^31−33^*In situ* experimental observation of the iron loading into ferritin not only enables an in-depth study of iron-related diseases but also provides insights into their potential cures. The ability to monitor the structural dynamics of ferritin during iron loading holds the potential for discovering innovative strategies for employing apo-ferritin as a natural drug delivery carrier.

Figure 1 shows the experimental setup utilized to capture and interrogate individual ferritin proteins in different liquid environments. This high-precision setup enables trapping and monitoring of a single ferritin, in real time. The DNH structure introduces a tightly confined optical field (see the field distribution of different DNHs in Figure S2), which provides a sufficient gradient force to retain a single protein in the hot spot with the nanoscale area.^28^ The intensity of the light transmitted through the DNH structure is measured by an avalanche photodiode (APD) in real time. The optical signal detected by APD reveals the conformations of the proteins as well as the trapping stiffness that is affected by the size of the molecule.^29^ After trapping individual proteins, the designed microchannel flow system introduces different solutions to observe their influence on the protein conformation. See Materials and Methods (section SI-1) in the Supporting Information for more details.

**Figure 1:**
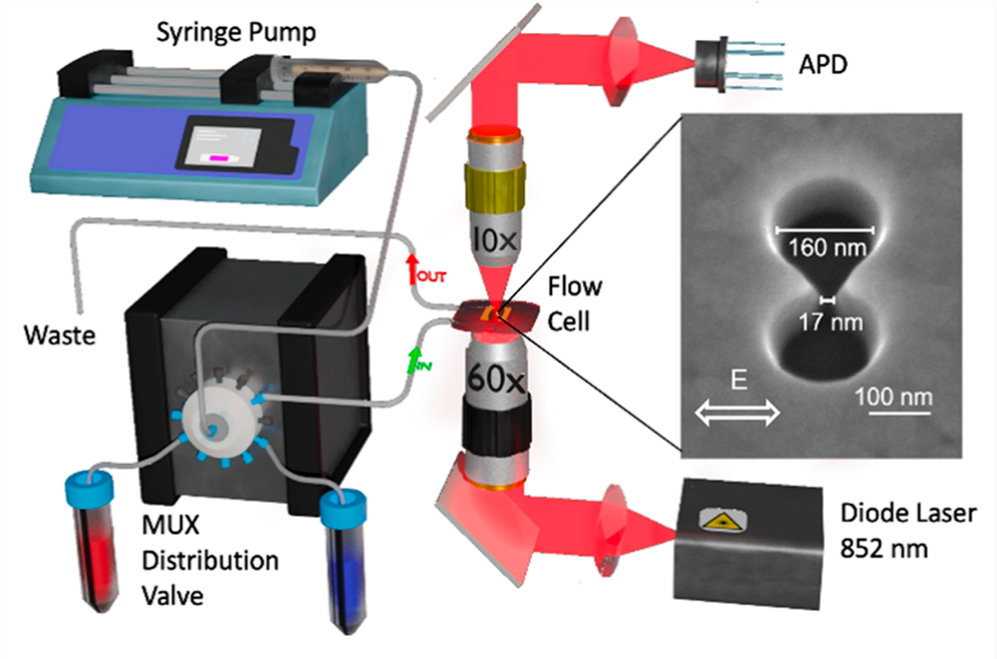
Schematic of optical nanotweezer setup. A syringe pump withdraws different buffers through a 12-way valve and then injects the buffer into the flowcell with controllable flow rates. The fused silica chip containing a double-nanohole (DNH) in a thin gold film is mounted in the flowcell. A collimated laser beam with a wavelength of 852 nm is focused on the DNH by a 60× air objective with NA 0.85. The transmitted light is collected by a 10× objective and then detected by an avalanche photodiode (APD). Right inset: scanning electron microscopy (SEM) image of a DNH structure in a 100 nm gold film, taken at an angle of 20° above the plane.

Once a dielectric object, i.e. ferritin in this case, enters the hot spot of the DNH, the transmitted intensity through the DNH changes, as the refractive index of the protein is higher than the environment around the trapping site; this effect is known as dielectric loading.^28,34,35^Figure 2a,b demonstrates the resolution of the developed setup, capable of detecting and distinguishing an apo-ferritin (Figure 2a) and a holo-ferritin (Figure 2b) by the transmitted optical signals of the DNH structure. In this work, the typical wait time for a DNH to trap a ferritin protein is 10–15 min (Figure S8). We trapped apo-ferritin and holo-ferritin using six DNH structures (#1, #2, #3, #4, #5, #6) that were fabricated with the same FIB parameters (Materials and Methods (section SI-1) in the Supporting Information), and each time the same structure was used for trapping both apo and holo proteins. The results demonstrated in Figure 2a,b were obtained by using DNH #3. The trapping signal from the same structure is generally consistent when acquired within a narrow time frame (see Figure S6). The transmission signal of the DNH with a trapped apo-ferritin (Figure 2a) exhibits fluctuations with a larger magnitude compared to that of holo-ferritin (Figure 2b). This observation of a stable optical signal of holo-ferritin at the single-protein level agrees well with the previous anticipations that the “holo” form of ferritin is more rigid than its “apo” form.^36,37^ To the best of our knowledge, this is the first experimental demonstration of the difference in structural rigidity of native ferritins at the individual protein level.

**Figure 2:**
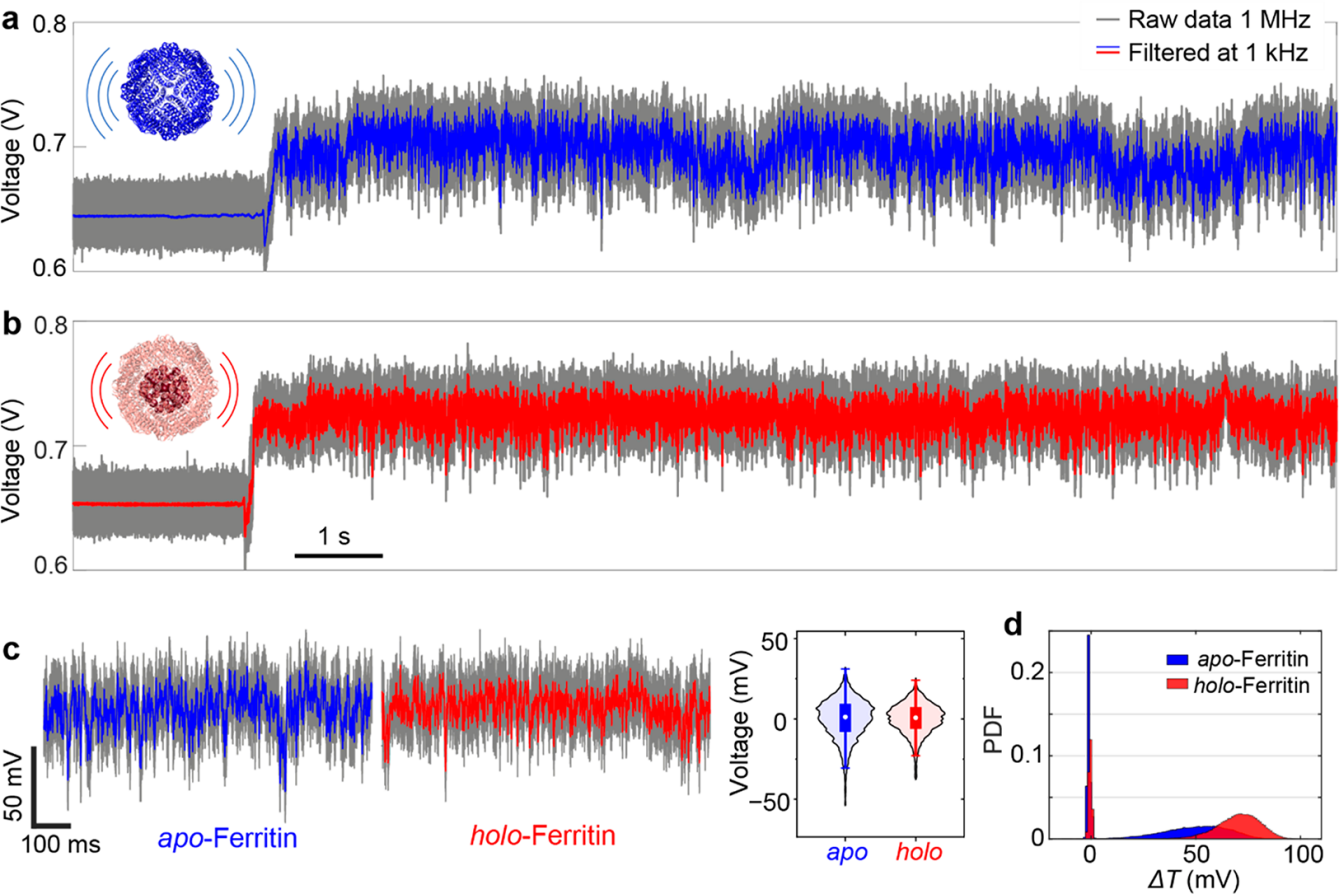
Optical signals from the DNH reveal the difference in structural dynamics between apo- and holo-ferritin at a single-molecule level. (a, b) Transmission signal through the same DNH upon trapping a single apo-ferritin (a, blue) and holo-ferritin (b, red) for 12 s. Insets: crystal structure of apo-ferritin (PDB: 2W0O) and holo-ferritin (PDB: 6TRZ) with curved lines indicating their structural fluctuation in the trap. (c) (left) 1 s magnified trace from (a) and (b) normalized to the transmission value with the highest probability of each trace. (right) Box plot indicating the interquartile range along with the violin plot of two 1 s traces filtered at 1 kHz. (d) Probability density function (PDF) of the transmitted optical signal calculated from 12 s traces of trapping an apo-ferritin ((a) blue) and a holo-ferritin ((b) red). All of the traces were digitally filtered with a cutoff frequency of 1 kHz. Data were acquired at 1 MHz.

The difference in structural dynamics of apo- and holo-ferritin is further proven by the comparison between the 1 s transmission signal of DNH with the presence of an apo-ferritin (blue) and holo-ferritin (red) in Figure 2c. The transmission intensity of apo-ferritin reveals a wider distribution compared to that of holo-ferritin (left panel of Figure 2c), indicating a less stable structure of apo-ferritin. This result is further confirmed by the kernel density distribution of two traces filtered at 1 kHz, along with the box plot indicating the interquartile range in the right panel of Figure 2c.

Figure 2d shows the probability density function (PDF) of the changes in transmission signal through a DNH (Δ*T*) before and after trapping a single apo-ferritin and holo-ferritin. Trapping a holo-ferritin (red) leads to a transmission change with a larger amplitude compared to its apo counterpart. We attribute this large change in transmission signal to the increased polarizability of holo-ferritin due to its ferrihydrite core. First, the conductivity of the protein increases because the ferrihydrite core provides two tunnel barriers by the protein shell.^19^ According to the Drude model,^38^ the polarizability of a particle increases with its conductivity. Second, the size of the particles directly impacts their polarizability.^39−41^ Several research works anticipated the difference in the average size of apo-ferritin and holo-ferritin, with holo-ferritin being about 2 nm larger than the apo form.^17,42,43^ This larger size and increased conductivity of a single holo-ferritin are clearly detected through a higher Δ*T*/*T*_0_ in the optical signal, due to its higher polarizability. Table S1 gives the data related to Δ*T*/*T*_0_ of the proteins, measured by all six sets of DNH structures.

To examine the reliability of the technique presented here, we have repeated the experiments with 6 different DNH structures fabricated with the same parameters (see SEM images in Figure S1 and trapping traces in Figure S3). Figure 3 summarizes the normalized root-mean-square (NRMS) and the PDF of the normalized voltage, both associated with fluctuations in the transmission signal when individual ferritins are trapped. We note that grain in the gold film and/or the condition of FIB led to different geometries of DNHs as shown in Figure S1c. The results in Figure 3 demonstrate that the difference in trapping dynamics between apo- and holo-ferritins is regardless of potential geometrical variation or resonant behavior of DNH structures.^26,44^ On the other hand, the DNH structure loses its effectiveness in capturing proteins after repeated uses (Figure S1); therefore, all the experiments were acquired within 2–3 weeks with 4–5 h of laser illumination.

**Figure 3:**
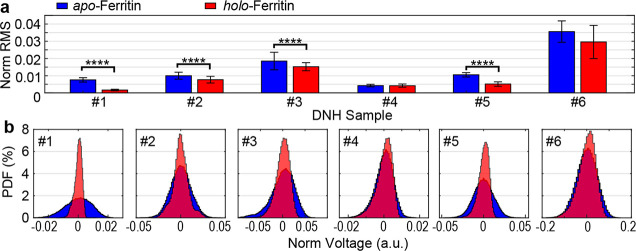
Comparison of the optical trapping signal of individual apo-ferritins and holo-ferritins. (a) Normalized root-mean-square (NRMS) of transmission signal when an apo-ferritin (blue) and a holo-ferritin (red) are trapped in six different DNH structures. (b) PDF of 20 s traces with apo-ferritin (blue) and holo-ferritin (red) trapped in six different DNH structures. Data were acquired at 1 MHz and then Gaussian filtered at 1 kHz. Asterisks indicate statistically significant differences (****p < 0.0001).

Previous work reported that the hydrodynamic movement of the particles in a harmonic trap leads to a linear correlation between protein molecular weight and the RMS of the trapping signal.^45^ Trapping a holo-ferritin is therefore expected to produce a larger NRMS due to its larger molecular weight compared to apo-ferritin. In our experiment, out of the six DNH structures, however, four (#1, #2, #3, #5) reported significantly smaller RMS values related to holo-ferritins (*p* value <0.0001, Figure 3a). This observation suggests that in addition to the hydrodynamic movement, apo-ferritin exhibits large-scale conformational fluctuations at a relatively low frequency (3–150 Hz, see Figure S5 in section SI-4 in the Supporting Information) that are captured by the optical signal.^45^ The iron binding to the negatively charged interface leads to the decreased overall motion of holo-ferritin,^36^ resulting in a stabilized conformation and thus a lower NRMS in the trapping signal. This RMS from the optical signal is similar to the root-mean-square deviation (RMSD) in molecular dynamics simulations, which inversely correlates with protein stability.^46^

The experimental results in Figures 2 and 3 demonstrate a reliable technique for distinguishing single apo- and holo-ferritin based on their structural dynamics. Subsequently, in Figure 4 we demonstrate the exciting capability of this technique to monitor the global conformational changes of apo-ferritin during the process of iron ion loading and connect the structural dynamics to the folding and unfolding of the pore channels. In this experiment, we monitored the transmission signal of trapped ferritin in response to Fe^2+^ introduced by the fluidic system. The 30 min transmission trace of a single apo-ferritin trapped in a DNH (Figure 4a) along with the expanded views (Figure 4b–e) reveals the *in situ* iron loading process. After trapping the apo-ferritin in the DNH structure, we replaced the solution with a ferrous solution containing 2 mM Fe^2+^ ions at a flow rate of 4.5 μL/min. The arrows in Figure 4a mark the time of protein trapping, iron injection by the flow system (flow rate changed from 0 to 4.5 μL/min), before the ferrous solution reaches the protein (Figure 4b) and after the protein was exposed to the ferrous solution for different times (Figure 4c–e). Before the ferrous solution reaches the protein, the trapping signal of apo-ferritin in Figure 4b exhibits a relatively stable magnitude of fluctuations in the signal, consistent with the observation in Figure 2a. After the ferrous solution has arrived at the trapping site (Figure 4c,d), we observed nonuniform patterns in the transmission signal with some segments (purple) having reduced fluctuations.

**Figure 4:**
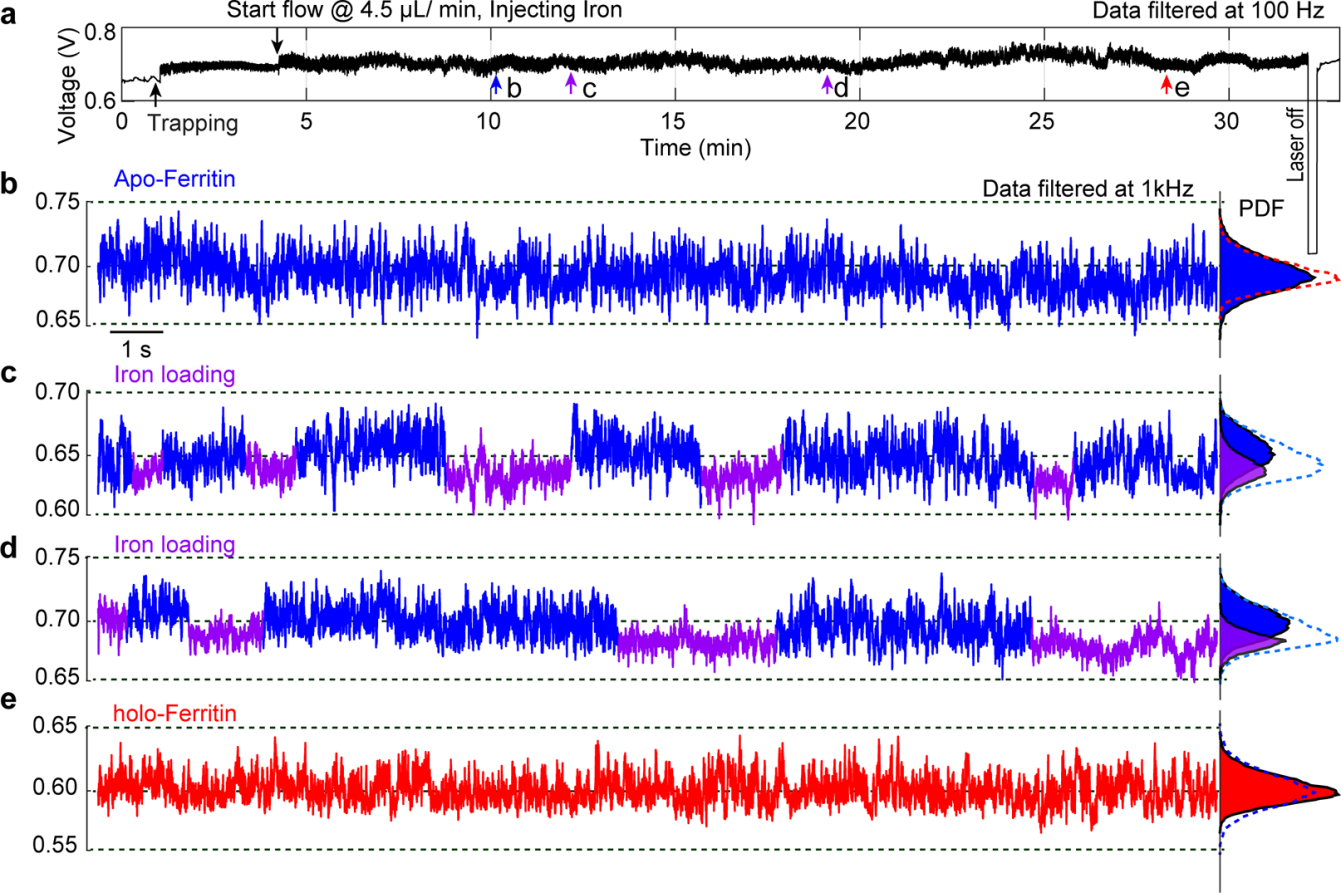
*In situ* iron loading into a trapped apo-ferritin. (a) Continuous transmission trace of a single apo-ferritin trapped in the hot spot of a DNH and then exposed to the ferrous solution for more than 20 min. After turning off the laser for 5 s, the transmission signal returned to the baseline, indicating that protein was released. (b) 20 s transmission trace of trapped apo-ferritin before the ferrous solution reaches the hot spot, along with the PDF of the trace on the right (blue). The red dashed curve represents the PDF of (e) for comparison. (c, d) 20 s transmission traces after apo-ferritin was exposed to the ferrous solution. The segments with lower RMS are colored in purple, which is identified by RMS changes as discussed in section SI-8 in the Supporting Information. The PDF plot on the right shows the PDF of the purple segments and blue segments, respectively, as well as the PDF of the whole trace (cyan dashed curve). (e) 20 s transmission trace after apo-ferritin was exposed to the ferrous solution for more than 20 min, along with the PDF of the trace (red) and the PDF of (b) (dashed blue).

We attribute these “on–off” (marked as blue-purple) patterns in the signal to the folding of 8 gated pores (3-fold channels) in the ferritin formed by the assembly of 24 subunits. The main passageway for iron ion transport through ferritin is via these 3-fold channels lined with the polar side chains aspartate and glutamate, which make the channel hydrophilic.^22,47^ The apo-ferritin is dynamically unstable due to the unfolding of the ion channels, resulting in large-magnitude fluctuations in the trapping signals (blue segments); these channels fold upon Fe^2+^ binding to all available sites, so the protein becomes more rigid, producing reduced fluctuations in the trapping signal (purple segments). After the initial oxidation of Fe^2+^ at the ferroxidase centers, the resulting Fe^3+^ is transferred to the protein cavity by the 3-fold channels. These channels unfold again when the transfer is finished; therefore, the protein is relaxed again, resulting in increased fluctuations in the trace (blue segments). The repetition of the above activities produces the “on–off–on” patterns in the trapping signal.

We highlight that the average time of the on–off cycle measured here is comparable with the binding times previously reported via bulk measurements. As reported previously, when the number of ions occupies all metal binding sites of the ferritin, it takes 10–20 s for the metal to transport to the ferroxidase centers, where it binds and is oxidized to Fe^3+^.^47^ The trapping traces in Figure 4c,d suggest that the dwell time for on and off states range from 2 to 5 s. It is worth mentioning that the dwell time changes between individual ferritin proteins, as repeated experiments conducted by other DNH structures revealed different dwell times of about 20 s (Figure S10a,b). Moreover, repeating on–off patterns induced by ferroxidase pore gating (conformational change) not only lets more Fe^2+^ ions enter the protein cage but also assists the translocation of Fe^3+^ from the ferroxidase center into the internal cavity of the ferritin.^3,48^ After 22 min of iron flow to the trapping site, the amplitude of the signal decreased with reduced fluctuations (Figure 4e). The median RMS decreased from 6.8 to 5.4 mV (see Figure S11), indicating that the protein becomes more rigid upon iron mineralization in the protein core, consistent with the observation in Figures 2 and 3.

On the other hand, previous research using GLC-TEM has shown that the iron oxide core starts to form inside the apo-ferritin after 1 h of biomineralization.^17^ As marked by the red arrow in Figure 4a, our single-molecule data indicate that this biomineralization takes approximately 20–30 min instead of 1 h. This relatively short process may be due to the high temperature (∼49.8 °C) in the trapping site induced by laser heating (Figure S12), which is also lower than the 56 °C temperature where ferritin pores have been observed to melt.^46^ In addition, the global structure of ferritin is stable up to 85 °C;^46^ therefore, the laser-induced heating will not affect the overall structure of the ferritin. We note that the ferrous solution is continuously delivered to the protein site to ensure the ratio of Fe^2+^ to ferritin is larger than 200:1.^49,50^ Control experiments performed by injecting PB buffer containing Fe^3+^ (instead of Fe^2+^) into a DNH with a trapped apo-ferritin did not result in the on–off pattern (Figure S13). Moreover, the median RMS of a single apo-ferritin after 20 min of exposure to the ferric solution (15 mV) is roughly the same as that before the exposure to this solution (14 mV) (Figure S14). This result confirmed that the on–off patterns in Figure 4 are associated with Fe^2+^ loading.

In conclusion, we demonstrated the first experimental evidence of the dynamic difference between individual, unlabeled apo- and holo-ferritins via an optical nanotweezer system. By employing this high-precision optical characterization setup, we have not only managed to differentiate apo- and holo-ferritin at a single-molecule level but also monitored the real-time dynamics of a single apo-ferritin converting into a holo-ferritin noninvasively. In detail, we demonstrated that the optical trapping signals from the same DNH but different trapped proteins (i.e., an apo- or a holo-ferritin) provide two parameters to discriminate the isoforms of ferritin. (1) Holo-ferritin produces a lower RMS of the trapping signal compared with its apo counterpart, in line with the relatively stable structure of holo-ferritin. (2) Trapping a holo-ferritin introduces a 41% larger change on average (Table S1) in the transmission signal compared to that of an apo-ferritin, indicating a higher polarizability due to the larger size and higher conductivity of holo-ferritin. In addition, this work provides the first experimental evidence of *in situ* iron loading into a single, unmodified apo-ferritin molecule. By analyzing the transmission signals, we managed to track the structural dynamics of ferritin associated with the gating behavior of the 3-fold channels. These 3-fold channels undergo unfolding (on) and folding (off) cycles, to let the Fe^2+^ enter the ferroxidase centers (unfolding), and to transfer the Fe^3+^ to the protein core (folding). Monitoring the unfolding and folding cycles of pore channels associated with biomineralization of iron ions inside the ferritin opens up the potential of controlling the protein cages precisely to deliver essential medicines to living systems and for metal nanoparticle encapsulation.

Recently, scientists have been developing label-free single-molecule approaches to overcome the challenges of studying proteins. Pioneering techniques, such as interferometric scattering microscopy (iSCAT)^51^ and nanopore electro-osmotic trap (NEOtrap),^52^ have demonstrated their potential in sizing proteins and monitoring protein dynamics but so far are limited to proteins larger than 50 kDa.^53^ The DNH-based protein trapping allows the detection of small proteins down to 6.5 kDa.^45^ Therefore, the technique presented in this work may be extended to monitoring the pore-gating dynamics of wide-range-sized proteins whose functions are dependent on their globular conformations.

## Supporting Information Available

Materials and methods, characteristics of DNH structures and their influence on protein trapping, trapping individual apo- and holo-ferritins by other DNH structures, noise of the same DNH in different scenarios, change in the transmission signal upon trapping single proteins, reproducibility and consistency of the trapping signal, wait time for DNHs to trap a single protein, identification of low RMS segments in the trapping traces, repeat experiments of *in situ* iron loading on a single apo-ferritin, change in the apo-ferritin single-molecule dynamics upon iron mineralization, temperature in the trapping site, and exposure of the single apo-ferritin to Fe^3+^ (control experiment for iron loading) (PDF)
