# Supplementary material for: Optical Monitoring of In Situ Iron Loading into Single, Native Ferritin Proteins
Source: Nano Lett. 2023 Apr 13;23(8):3251–8. doi: 10.1021/acs.nanolett.3c00042 (PMC10141409; doi:10.1021/acs.nanolett.3c00042)
Supplement: Supplementary file 1 — nl3c00042_si_001.pdf [file nl3c00042_si_001.pdf]

Supporting Information

Optical monitoring of *in-situ* iron loading into single, native ferritin proteins

Arman Yousefi[1], Cuifeng Ying[1], Christopher D. J. Parmenter[2], Mahya Assadipapari[1], Gabriel Sanderson[1], Ze Zheng[1], Lei Xu[1], Saaman Zargarbashi[1], Graham J. Hickman[3], Richard B. Cousins[2], Christopher J. Mellor[4], Michael Mayer[5], and Mohsen Rahmani[1*]*

[1] Advanced Optics and Photonics Laboratory, Department of Engineering, School of Science and Technology, Nottingham Trent University, Nottingham NG118NS, United Kingdom

[2] Nanoscale and Microscale Research Centre, University of Nottingham, Nottingham NG7 2RD, United Kingdom

[3] School of Science and Technology, Nottingham Trent University, Nottingham NG11 8NS, United Kingdom

[4] School of Physics and Astronomy, University of Nottingham, Nottingham NG7 2RD, United Kingdom

[5] Adolphe Merkle Institute, University of Fribourg, Chemin des Verdiers 4, CH-1700 Fribourg, Switzerland.

Corresponding Authors Email: cuifeng.ying@ntu.ac.uk; Mohsen.rahmani@ntu.ac.uk

SI-1 Materials and Methods

Fabrication of Nanostructures on Gold Thin Film: The double nanohole (DNH) structure used through this work was fabricated based on that reported by Ying et al.[1] Accordingly, 550 μm thick fused silica wafers were coated with a 30 nm silicon nitride (SiN_x) layer using low-pressure chemical vapour deposition (LPCVD) at 800˚C. The resulting silicon nitride layer was then covered with a 5 nm thick Ti film, as an adhesion layer, followed by a 100 nm thick Au film, both of which were deposited using an electron beam evaporation (Leybold Optics LAB 600H) at 190°C. Afterwards, by using a dicing machine (Disco DAD321) the silica wafers were diced into 10 mm × 10 mm chips. To create the double nanohole (DNH) structures in the gold film, we used a focused ion beam (FIB, Zeiss Crossbeam) with a gallium ion source. We created the DNH structure using the geometry that consists of two circles (Φ 160 nm) with a

centre-to-centre distance of 200 nm. A rectangle (3 nm × 40 nm) is placed in the middle of the two circles to bridge them. To create the DNH structure, we ran the focused ion beam (FIB) at an ion beam energy of 30 kV, and a beam current of 1 pA. The dwelling time for making circles was 1.25 μs, and for the box was 5 μs. These parameters were optimised so that the gap sizes of the DNH structures are mostly around 20 nm with no gold residuals inside the gap. Using scanning electron microscopy (SEM) in top-view and tilted modes, we captured images of the DNH structures (Figure 1, and Fig. S1).

Optical Tweezer setup: All the optical components were acquired from Thorlabs as described previously.[1] The laser was focused into a spot with a diameter of about 1.2 μm using a 60× Plan Fluor objective with a numerical aperture (NA) of 0.85 (Nikon, Tokyo, Japan). A half-wave plate adjusts the polarisation of the laser to be perpendicular to the line that connects the centres of two holes.[2,3] The power density at the DNH sample was 19 mW/μm^2 due to the 32 mW incident laser power prior to the objective. The intensity of the transmitted light was detected by a silicon avalanche photodiode (APD120A, Thorlabs), which transformed the light intensity into a voltage signal. The voltage signal of the APD was recorded with a data acquisition card (USB-6361, NI) at a sampling rate of 1 MHz using a customised LabVIEW program.

Preparation of Protein and Iron Solutions: Both apo-ferritin (from equine spleen, A3660) and holo-ferritin (from equine spleen, F4503), as well as other chemicals, were purchased from Sigma-Aldrich, United Kingdom. We used 0.5 μM apo-ferritin or 0.5 μM holo-ferritin in 0.1 M phosphate buffer (PB, pH 7.4) for

the trapping experiments. To make a 2 mM ferrous solution, we first made 2 mM $Na_2S_2O_4$ in the PB buffer to deoxygenate the media.[4] Then we added Ammonium iron (II) sulphate to this solution and magnetically stirred the mixed solution for about 10 min. For the PB buffer containing Fe^{3+} that was used for the control experiment, we prepared the 2 mM ferrous solution, but this time we bubbled the solution with compressed air for at least five minutes to oxidise Fe^{2+} to Fe^{3+}. We observed an obvious colour change from green to yellow after bubbling.[5] All the solutions were prepared freshly and were filtered through a 0.22 μm pore size filter before every experiment.

Fluidic system: The flow cells used in this work are the same as previously reported.[1] We print the flow cells by using the FormLab 2 printer with Clear V4 resin at a resolution of 50 μm (Formlabs Inc., USA). A two-component silicone-glue (Twinsil, Picodent, Germany) was used to seal the samples in the flow cell with a cover glass with a thickness of 0.17 mm. The DNH sample and the cover slide were separated by a double-sided tape with a thickness of 50 μm (ARcare92712, Adhesive Research, Inc.), creating a fluidic channel with a volume of 3.5 μL. Through a 12-port valve (Mux Distrib, Elve Flow, France), a syringe pump (Harvard Apparatus, US) controlled the flow rate and flow direction. To get the buffer exchanged after trapping a protein, we infused the buffer into the flow chamber at a flow rate of 4.5 μL/min. According to the internal diameter of the tubing used in the flow controller system, the ferrous solution arrives at the flow chamber after 25 μL (6 min after injection) of the solution passing through the flow controller.

Data Analysis: We used MATLAB scripts to analyse all the data presented in this work. All raw data were filtered using a zero-phase Gaussian low-pass filter to the desired cut-off frequency (1 kHz or 5 kHz) by using the **filtfilt.m** function. We calculated the probability density functions (PDF) by using the **ksdensity.m** function. To compare two trapping signals, we aligned the signal by subtracting the trace from its median value (Fig. 2c). All the normalised RMS (Figure 3) were calculated by dividing the standard deviation of 1-s trace by its mean value.

SI-2 Characteristics of DNH structures and their influence on protein trapping

The DNH structure loses its effectiveness in capturing proteins after repeated uses, i.e. longer waiting time and shorter duration of trapping.[6] We attribute it to the rounded cusps and edges of DNH after use, which can be affected by the buffer and the laser power used in the experiments.

Figure S1a provides the SEM images of six DNH structures used in this work, imaged before (only #2, #3 and #6 available) and after trapping experiments. In addition to the contaminations on the surface, the edges and cusps became smooth, and the gap sizes increased after being used for trapping. The gap sizes of #2, #3, and #6 changed from 15, 17, and 25 nm to 28, 34, and 37 nm, respectively (black circles in Fig. S1b). The boxplot in Fig. S1b takes account of the gap sizes measured from all DNHs, including the structures that are not used in this work but were fabricated with the same parameters (SEM images in Fig. S1c). We

observed an increase in medium gap size from 24.4 nm to 35.6 nm after DNHs were used for two to three weeks, with a typical duration of 4-5 hours of laser illumination.

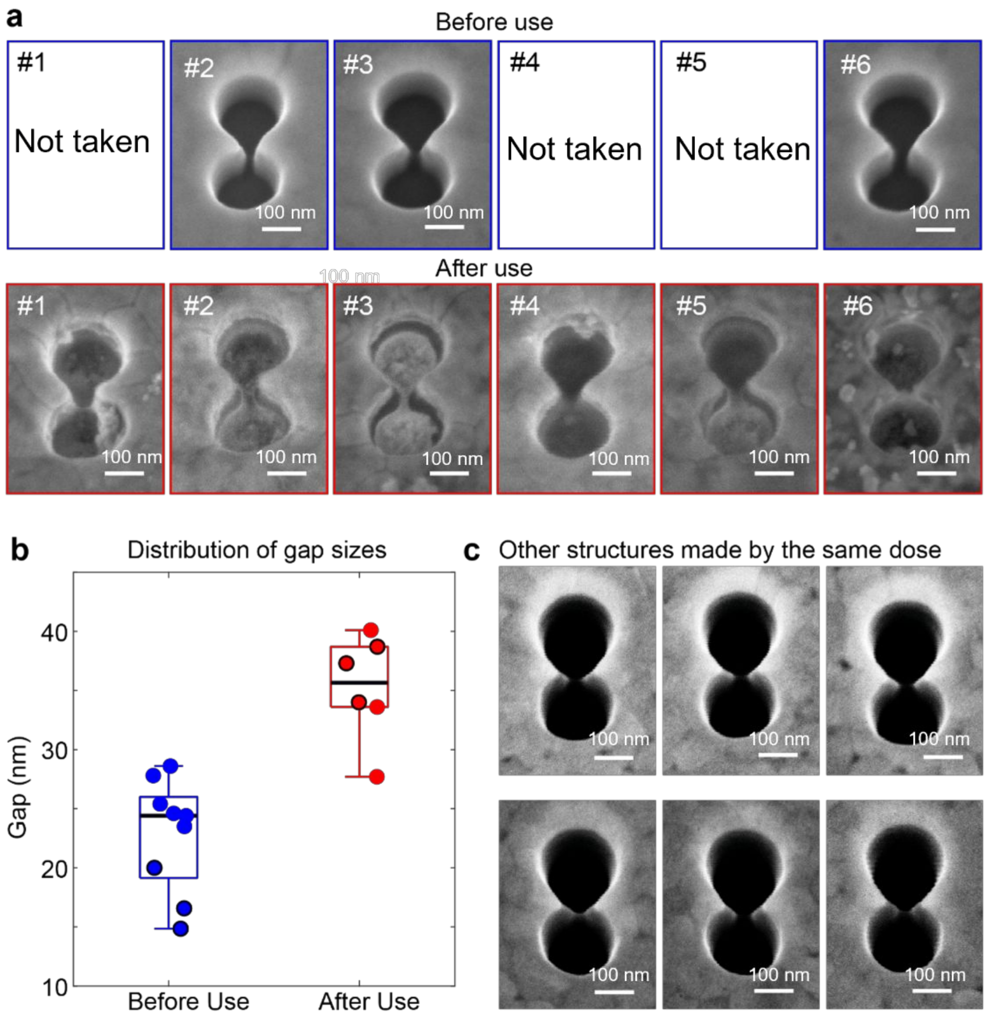

Figure S1. Slight variation in the the geometry of of DNH structures after trapping experiment. (a) SEM images of three DNHs (#2, #3, and #6) taken before use in the trapping studies. The SEM images of DNHs #1, #4, and #5 have not been taken. The SEM images on the bottom show all six DNH structures used after two to three weeks, with a typical duration of 4-5 hours of laser illumination. (b) Boxplot illustrating the

gap size distribution of DNH structures before and after use in the experiments. The data points with black circles represent the gap sizes of #2, #3, and #6. (c) SEM images of other unused DNH structures fabricated by using the same FIB parameters (see SI-1 Materials and Methods).

In order to understand the influence of gap size on the trapping efficiency, we simulated the optical field distribution of the DNH structures by using COMSOL Multiphysics 6.1 based on finite-element simulation. We consider an 852 nm laser with the polarisation perpendicular to the line that connects two holes. Figure S2a-c demonstrate the electric field distribution of three structures with gap sizes of 15 nm, 17 nm and 25 nm, representing #2, #3 and #6 respectively. As indicated by the scale of colour bars, the field enhancement decreases with increasing the gap sizes. Considering the dimension of the Ferritin protein (12 nm), we aim for DNH with a gap size of 20 nm to ensure the narrow trapping well at the same time allowing the protein to enter the hotspot. When the polarisation of the laser beam is parallel to the connecting line of two holes, as shown in Fig. S2d, little to no field enhancement was observed in the gap of DNH. This dependency on laser polarisation allows differentiating the DNH from other structures, as recently demonstrated.[7]

We note that the plasmonic optical trapping demonstrated here is based on the self-induced back-action, which operates well at the off-resonance wavelength. As shown in the transmission spectra in Figure S2e, the wavelength of the excitation laser at 852 nm is located at the off-peak of the localised surface plasmonic

resonance (LSPR) (at around 700 nm). Furthermore, we expect another resonance located in near-infrared wavelength as reported by Ghorbanzadeh et al.[3] Despite this off-resonance position, the electric field is still significantly enhanced in the gap of DNH. This off-resonance detection demonstrated the robustness of the SIBA trapping - without strict limitation on the excitation wavelength, the DNH structure can provide field enhancement that is strongly confined within the hotspot to trap single proteins.

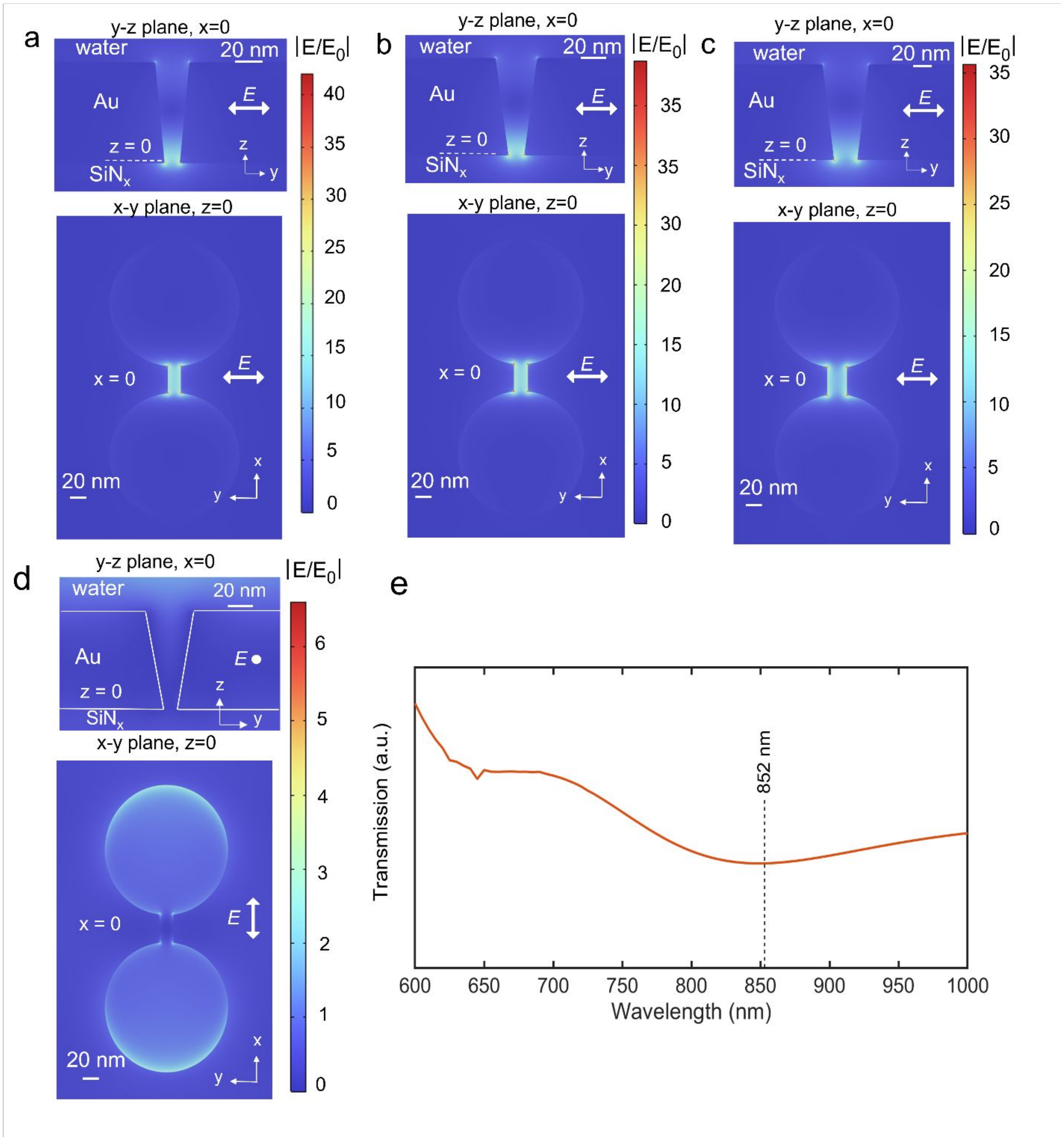

Figure S2. Simulated optical properties and distribution of field enhancement of DNH structures. (a-c), Electric field distribution within DNH structures with gap sizes similar to samples #2, #3, and #6. The top panels show the electric field distribution on the *y*-*z* plane at $x = 0$, and the bottom panels show the *x*-*y*

plane at $z = 0$ (gold-Ti interface). The incident laser is polarised along the y-axis (d) The top and bottom panel descriptions are similar to (a-c) but this time the polarisation of the incident laser is aligned to the x-axis. (e) Simulated normalised transmission spectra of the DNH structure with a gap size of 17 nm (#3) with laser polarisation shown in panel b.

SI-3 Trapping individual apo and holo-ferritins by other DNH structures

Due to the grains in the gold film and the variation of the FIB situation, different DNHs might have different features. For comparison between the dynamic of apo-ferritin and holo-ferritin, we trapped individual proteins in six different DNH structures and produced the RMS, and probability density function (PDF) shown in Figure 3. Figure S3 shows the trapping events of both apo-ferritin and holo-ferritin in five other structures (#1, #2, #4, #5, #6, and results of #3 are presented in Figure 2).

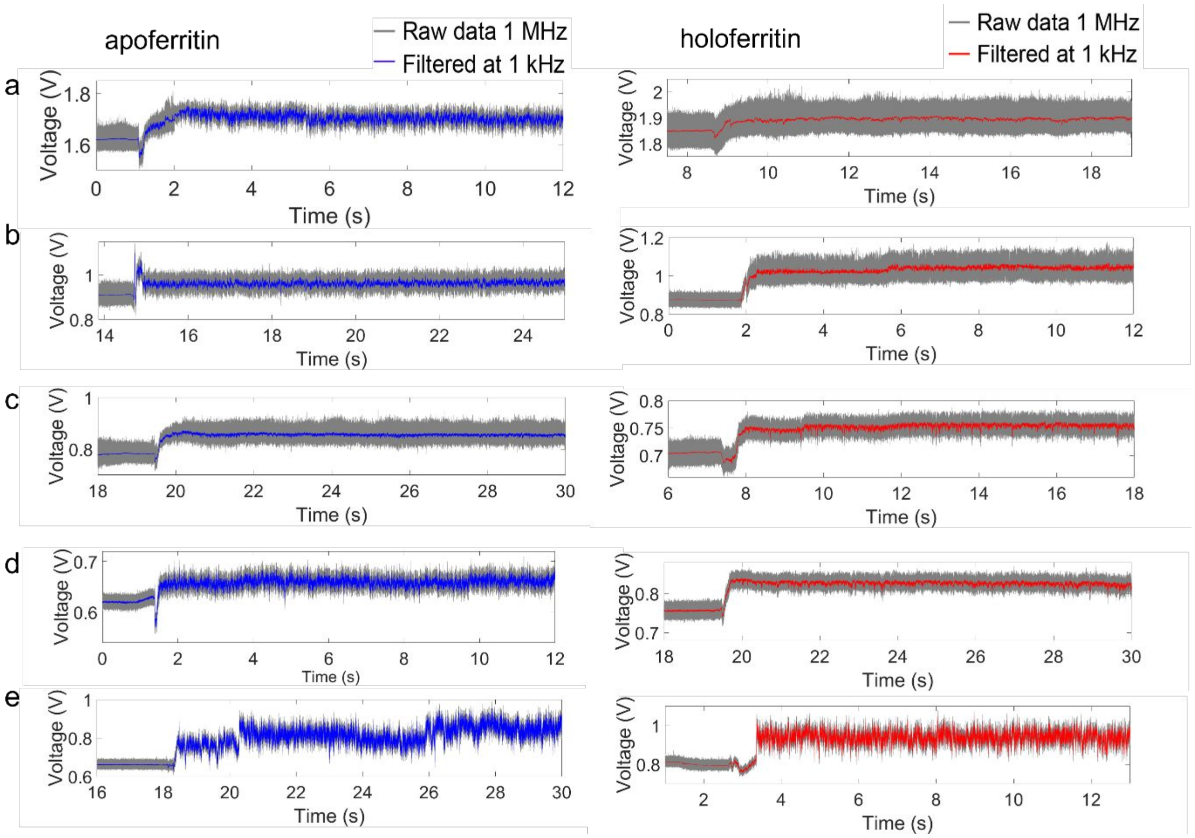

Figure S3. Five sets of transmission signals for single apo-ferritin and holo-ferritin trapped in the same DNH structures. (a, b, c, d, and e) are transmission signals of DNH structures #1, #2, #4, #5, and #6, respectively, upon trapping single apo-ferritin (blue traces) or single holo-ferritin (red traces) proteins. All the data were acquired at 1 MHz (grey) and then digitally filtered with a cut-off frequency of 1 kHz (blue and red).

SI-4 Noise of the same DNH at different scenarios

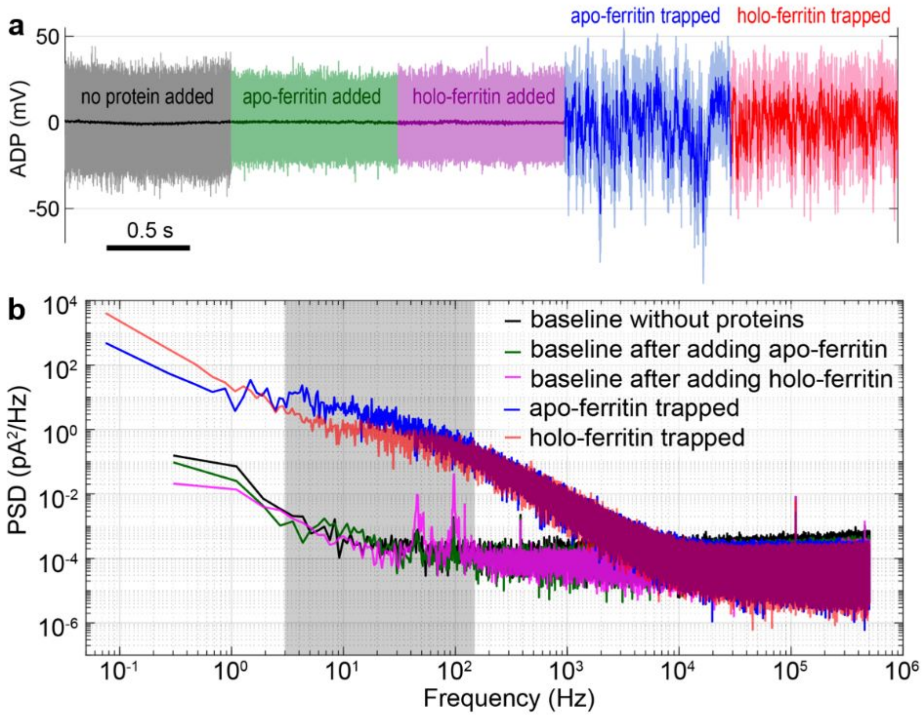

Figure S5. Comparison of noise at different frequency domains for the same DNH (#3) at different scenarios. (a) 5-second baseline before adding proteins, after adding apo- and holo-ferritin but no protein was trapped. Data are shown in raw (light coloured) and digital filtered at 1 kHz. (b) Power spectral density (PSD) of the baselines and transmission traces shown in panel a. The grey area highlights the frequency range where the transmission signal of the trapped apo-ferritin exhibits larger fluctuations compared to that of holo-ferritin. No significant differences were observed between baselines except that the line noise (50 Hz and 100 Hz) for baseline after adding holo-ferritin is slightly higher, possibly due to different alignments. Data were recorded at a sampling rate of 1 MHz.

SI-5 Change in the transmission signal upon trapping single proteins

Table S1 summarises the changes in the transmission signal of six DNH samples upon trapping apo-ferritin and holo-ferritin. Here, V_0 is the mean value of the APD signal for the empty DNH, V_1 is the mean value of the APD signal for the DNH with protein trapped. The changes in the transmission signal ($\Delta T/T_0$) were calculated by $(V_1 - V_0)/V_0$. Four out of six structures exhibit a higher $\Delta T/T_0$ value for trapping holo-ferritins than that for apo-ferritins.

Table S1. Change in the transmission signal of six different DNHs upon trapping apo and holo-ferritin proteins

DNH	Protein form	V_0	V_1	$\Delta T/T_0$
#1	Apo	1621	1715	0.057
	Holo	1838	1895	0.031
#2	Apo	909	960	0.056
	Holo	874	1040	0.189
#3	Apo	644	695	0.079
	Holo	653	724	0.108
#4	Apo	776	853	0.099
	Holo	704	752	0.068
#5	Apo	620	659	0.062
	Holo	755	825	0.092
#6	Apo	660	760	0.151
	Holo	813	939	0.154

SI-6 Reproducibility and consistency of the trapping signal

Figure S6 illustrates two consecutive trapping events of single apo-ferritin by using the same DNH structure (#2, other traces from this structure are shown in Fig. S3b). Both the RMS noise and $\Delta T/T_0$ demonstrate the reproducibility and consistency of the dynamic characteristics of identical proteins by using the same structure.

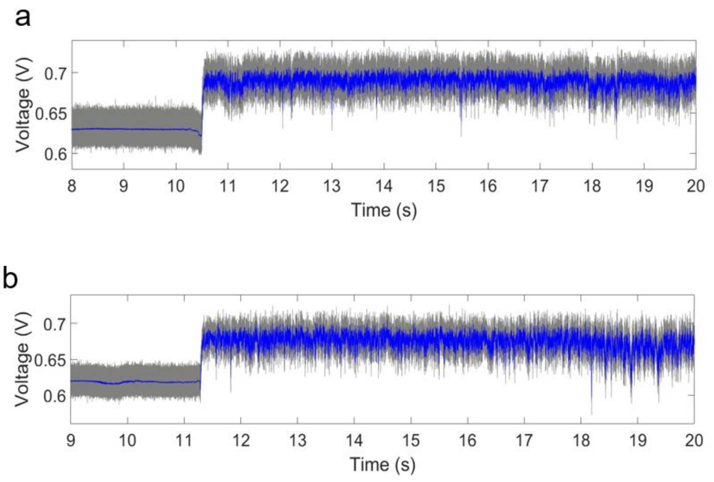

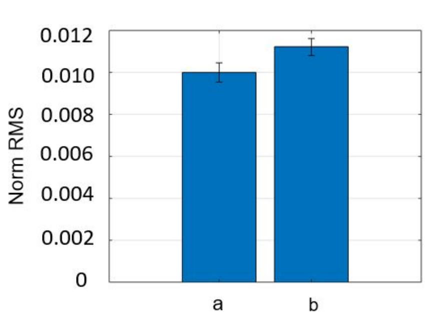

d

DNH	Trapping event	V_0	V_1	$\Delta T/T_0$
#2	a	629	689	0.095
	b	618	678	0.095

Figure S6. Comparison between two trapping events within the same DNH structure. (a) first trapping event and transmission signal of single apo-ferritin in DNH #2. (b) second single apo-ferritin trapping event and transmission signal 3 minutes after the first trap. (c) Comparison between normalised RMS of the two traces with apo-ferritin trapped. (d) comparison between the changes in the transmission signal ($\Delta T/T_0$) of two consecutive apo-ferritin trapping.

In this work, we observed mostly an increase in transmission upon trapping a single protein. Occasionally, trapping a protein induces a reduced transmission of DNH likely due to the protein being trapped in different trapping well (i.e. gold-water interface and gold-SiN_x interface). Figure S7 gives two "down-trapping" events of apo- and holo-ferritin by using the same structure (#4). The dynamic information carried by such "down-trapping" is consistent with the "up-trapping" events.[1] We observed a larger change in transmission signal ($\Delta T/T_0$) and a smaller RMS for trapping a holo-ferritin, compared to trapping an apo-ferritin.

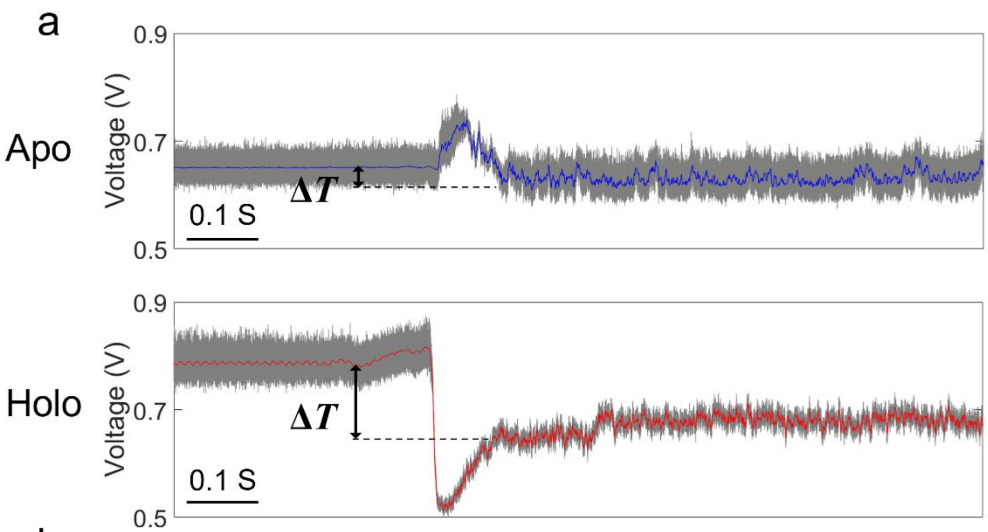

b

DNH	Protein form	V_0	V_1	$\Delta T/T_0$
#4	Apo	649	630	-0.029
	Holo	786	680	-0.134

Figure S7. (a) Optical transmission of a DNH upon trapping an apo- and holo-ferritin. (b) Comparison of the transmission change ($\Delta T/T_0$) of the trapping traces in panel a.

SI-7 Waiting time for DNHs to trap a single protein

We summarised the time duration between tuning on the laser and trapping of a ferritin protein. Figure S8 plots the waiting time of 25 trapping events from different DNHs. The histogram indicates that ferritin is most likely to be trapped within 10-15 min after the laser turns on. We note that the duration required to trap a single protein is impacted by a variety of factors, including the features of the (DNH) structure, the size of the protein being trapped, and the laser power used in trapping.[3] The surface repulsion between the negatively charged protein and the negatively charged gold surface can also lead to a longer duration needed for trapping a single ferritin.[6,8]

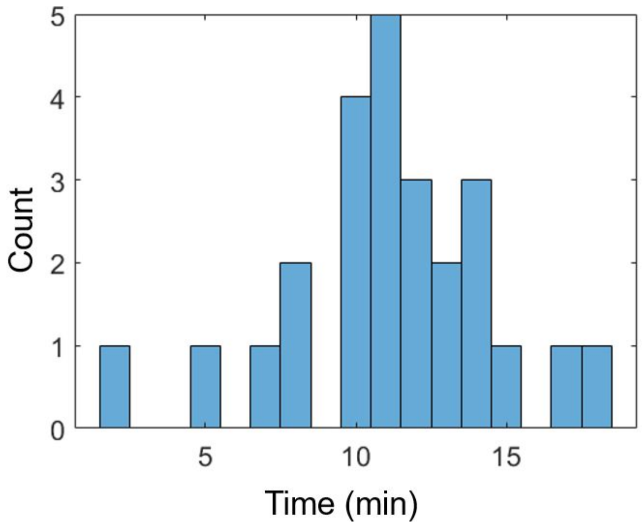

Figure S8. Histogram of the waiting time for trapping a ferritin protein after turning on the laser.

SI-8 Identify low RMS segments in the trapping traces

For identifying the low RMS segment in Figure 4, we created a MATLAB program to identify the step based on the changes in standard deviation (std). The red lines in Figure S9 represent stable levels with similar std for Figure 4b-4e of the main text. The threshold for step searching is 0.5 × max(std). We considered the levels that last more than 500 ms as the validated lower RMS segments. This algorithm identified and marked the lower RMS segments in purple segments (Fig. S9). We then manually connected some segments that are very close to each other.

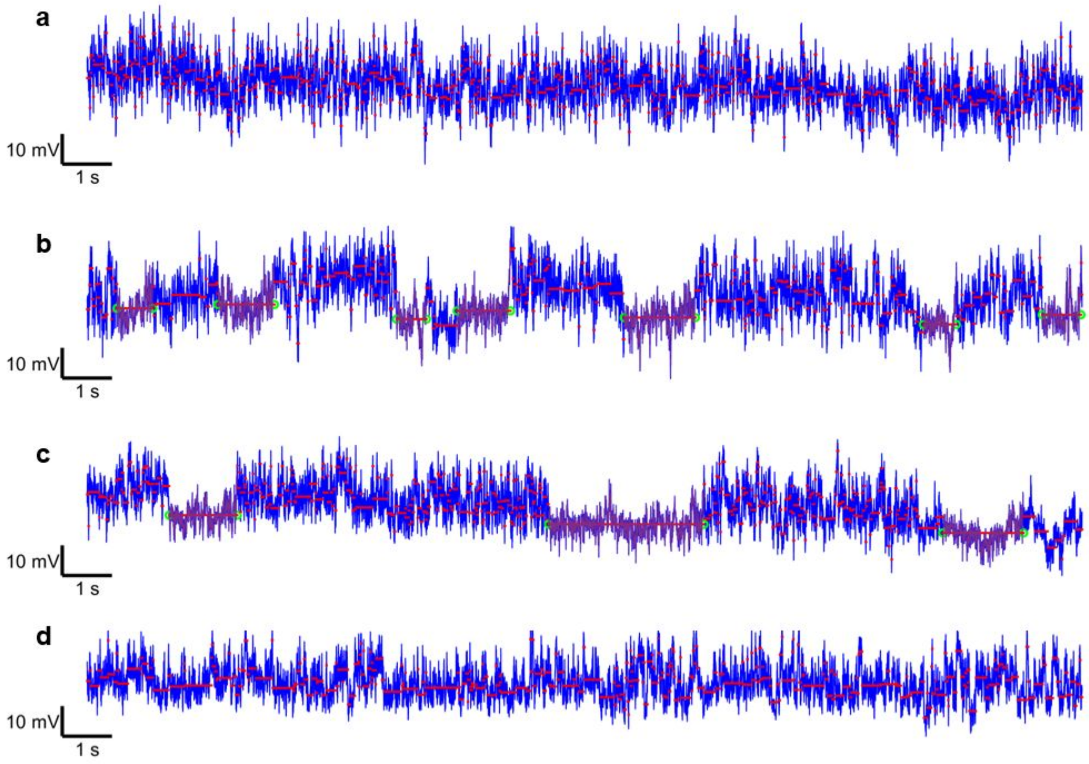

Figure S9. Level detection based on the change of standard deviation. The red lines with green circles on both ends are identified as lower RMS segments.

SI-9 Repeat experiments of in-situ iron loading on single apo-ferritin

We performed two more experiments to confirm the iron loading on single apo-ferritins. As expected, we observed some segments of reduced fluctuations in the transmission signal upon the iron solution arriving at the trapped apo-ferritin. Figure S10a illustrates a 120-s transmission signal of a DNH while a single apo-ferritin is trapped (top trace), and then in-situ loading iron into the protein (bottom trace). The duration of the channel folding during the Fe^{2+} entering the channels is 10-20 s (Fig. S10a-bottom trace - purple). Figure S10b (top trace) is a 20-s transmission signal of another trapped apo-ferritin in the same DNH structure. The duration of the channel folding is about 2-5 s (Fig. S10b bottom-purple).

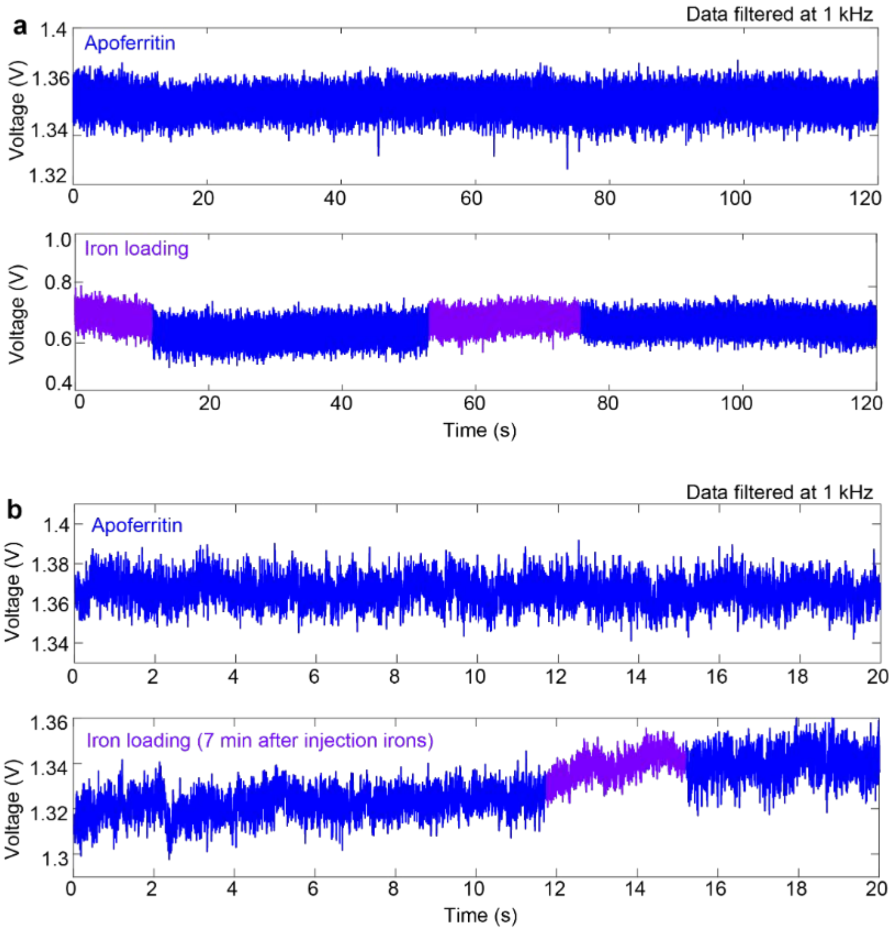

Figure S10. Two repeat experiments of iron loading on the single apo-ferritin. (a) Two minutes of transmission signal while a single apo-ferritin is trapped in the DNH structure (top trace). The trace on the bottom shows 2-min transmission signal after the apo-ferritin is exposed to the ferrous solution, resulting in (blue) – off (purple) patterns. (b) twenty seconds of transmission signal while a single apo-ferritin is trapped in the DNH structure (top trace). The trace on the bottom shows a 20-s transmission signal of apo-ferritin after being exposed to the ferrous solution, resulting in (blue) – off (purple) patterns. All data were acquired at 1 MHz and then digitally filtered with a cut-off frequency of 1 kHz.

SI-10 Change in the apo-ferritin single molecule dynamics upon iron mineralisation

Figure S11 shows the root mean square (RMS) of the transmission signal when a single apo-ferritin is trapped (blue) and after ~ 20 minutes of exposure to the Fe^{2+} solution. RMS decreases from 6.8 to 5.4 mV due to iron biomineralisation inside the protein cavity.

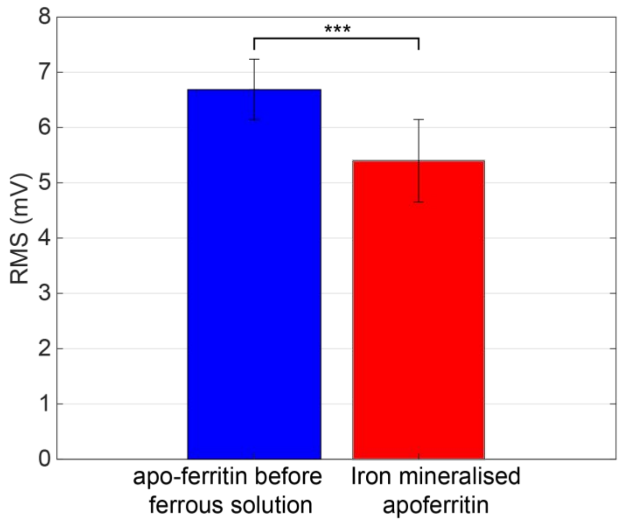

Figure S11 Root mean square (RMS) of the transmission signal when a single apo-ferritin is trapped (blue) and after 20 minutes of exposure to Fe^{2+} solution (red). The asterisks indicate that these two data sets are significantly different with a p-value of 0.0002.

SI-11 Temperature in the trapping site

Laser illumination on the gold nanostructures can induce a heating effect due to the photon absorption and subsequent energy dissipation. [9] We calculated the temperature in the trapping site by finite-element simulation in COMSOL Multiphysics.[10] Figure S12 shows the temperature profile of a double nanohole at the laser power of 32mW used in this work. Considering the best scenario, i.e., all the absorbed laser power is converted to heat, the temperature in the DNH structure is estimated to be 49.8 ˚C.

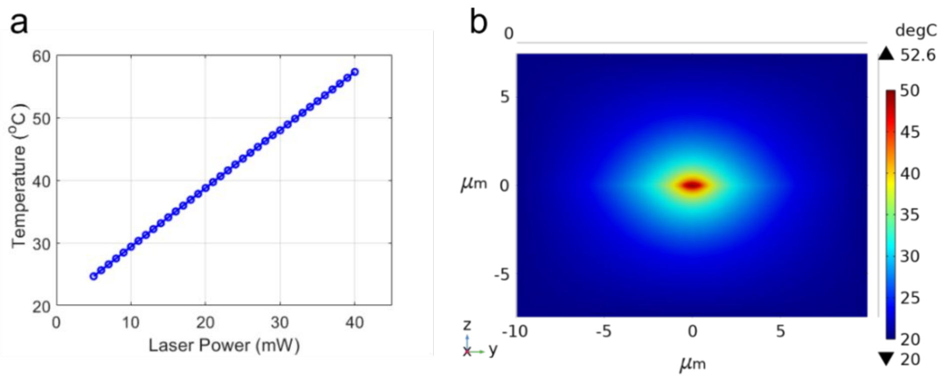

Figure S12. Simulation estimates of laser heating. (a) Finite-element simulation of the temperature in the DNH gap as a function of laser power. (b) Finite-element simulation of the temperature profile of the DNH structure when illuminated by a focused laser beam of 32mW.

SI-12 Exposing the single apo-ferritin to Fe^{3+} (control experiment for iron loading)

As the iron binding sites in ferritin bind only to ferrous (Fe^{2+}) ions[11], we performed a control experiment to expose apo-ferritin to ferric iron (Fe^{3+}) solutions. Figure S13a shows the transmission signal of the DNH with apo-ferritin trapped in the PB solution. We then replaced the solution in the chamber with a 2 mM ferric solution while the protein remained trapped. Figure S13b-S13d shows the optical trace after the apo-ferritin was exposed to the ferric solution for 8 min, 15 min and 20 min. No "on-off" patterns were observed. Figure S14 compares the RMS of the optical trace obtained before apo-ferritin was exposed to the ferric solution (trace shown in Fig. S13b) and obtained after the apo-ferritin was exposed to the ferric solution for 20 min (trace shown in Fig. S13e). No significant difference in the RMS between the two traces suggests

that apo-ferritin retained its relatively flexible conformation hence no iron loading happened.[12] Figures S13 and S14 again confirm that the "on-off" patterns observed in Figure 4 of the main text are due to the iron loading activity of ferritin.

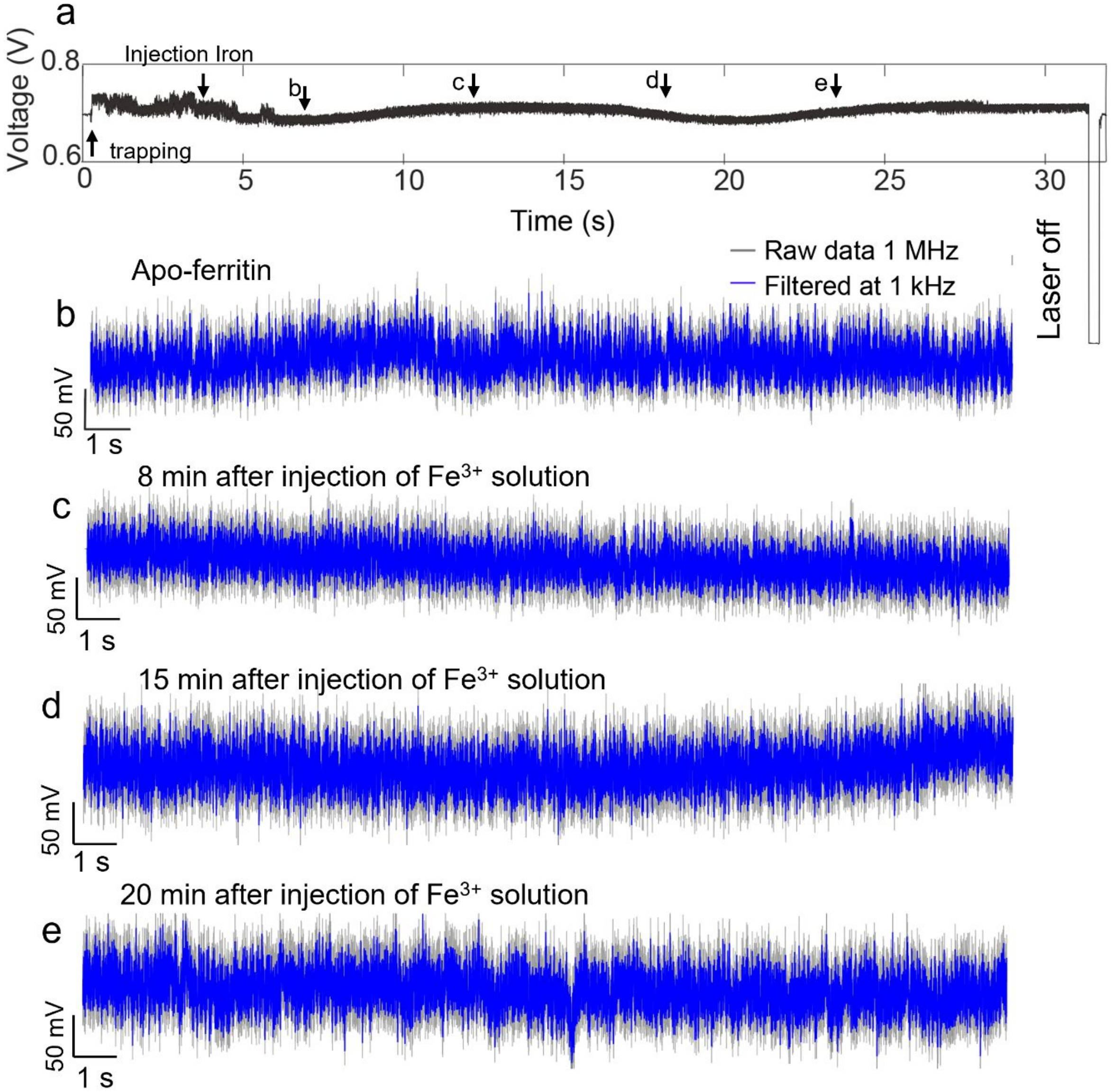

Figure S13. In-situ ferric iron loading into a trapped apo-ferritin, (a) Continuous transmission trace of a single apo-ferritin trapped in the hotspot of a DNH, then exposed to the ferrous solution for more than 20 min. After turning off the laser for 5 seconds, the transmission signal returns to baseline, indicating that protein was released. (b) 20-second transmission trace of trapped apo-ferritin before ferrous solution reaches the hotspot, (c and d) 20-second transmission traces after apo-ferritin was exposed to the ferric solution. (e) 20-second transmission trace after apo-ferritin was exposed to the ferric solution for more than 20 minutes.

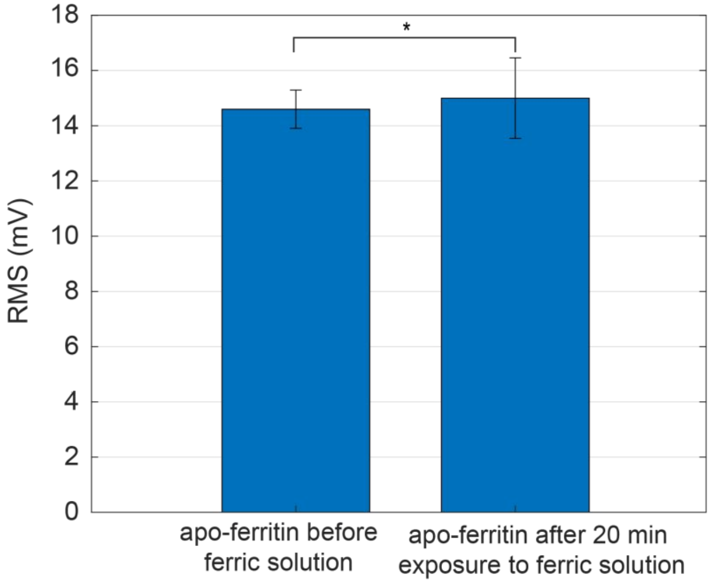

Figure S14. Root mean square (RMS) of 20 s transmission traces when a single apo-ferritin is trapped in PB solution and after it is exposed to Fe^{3+} solution for more than 20 min. There is no significant difference ($p = 0.119$) between these RMS results from the two traces.

References

(1) Ying, C.; Karakaçi, E.; Bermúdez-Ureña, E.; Ianiro, A.; Foster, C.; Awasthi, S.; Guha, A.; Bryan, L.; List, J.; Balog, S.; Acuna, G. P.; Gordon, R.; Mayer, M. *Watching Single Unmodified Enzymes at Work*. arXiv preprint arXiv:2107.06407. (accessed 2022-12-28).

(2) Pang, Y.; Gordon, R. Optical Trapping of 12 Nm Dielectric Spheres Using Double-Nanoholes in a Gold Film. *Nano Lett* **2011**, *11* (9), 3763–3767. https://doi.org/10.1021/nl201807z.

(3) Ghorbanzadeh, M.; Jones, S.; Moravvej-Farshi, M. K.; Gordon, R. Improvement of Sensing and Trapping Efficiency of Double Nanohole Apertures via Enhancing the Wedge Plasmon Polariton Modes with Tapered Cusps. *ACS Photonics* **2017**, *4* (5), 1108–1113. https://doi.org/10.1021/acsphotonics.6b00923.

(4) Boparai, H. K.; Comfort, S. D.; Shea, P. J.; Szecsody, J. E. Remediating Explosive-Contaminated Groundwater by in Situ Redox Manipulation (ISRM) of Aquifer Sediments. *Chemosphere* **2008**, *71* (5), 933–941. https://doi.org/10.1016/j.chemosphere.2007.11.001.

(5) Mungchamnankit, A.; Kittiauchawal, T.; Kaewkhao, J.; Limsuwan, P. The Color Change of Natural Green Sapphires by Heat Treatment. In *Procedia Engineering*; Elsevier Ltd, 2012; Vol. 32, pp 950–955. https://doi.org/10.1016/j.proeng.2012.02.037.

(6) Hacohen, N.; Ip, C. J. X.; Gordon, R. Analysis of Egg White Protein Composition with Double Nanohole Optical Tweezers. *ACS Omega* **2018**, *3* (5), 5266–5272. https://doi.org/10.1021/acsomega.8b00651.

(7) Ravindranath, A. L.; Shariatdoust, M. S.; Mathew, S.; Gordon, R. Colloidal Lithography Double-Nanohole Optical Trapping of Nanoparticles and Proteins. *Opt Express* **2019**, *27* (11). https://doi.org/10.1364/oe.27.016184.

(8) Gordon, R. Future Prospects for Biomolecular Trapping with Nanostructured Metals. *ACS Photonics* **2022**, *9* (4), 1127–1135. https://doi.org/10.1021/acsphotonics.2c00231.

(9) Jiang, Q.; Rogez, B.; Claude, J. B.; Baffou, G.; Wenger, J. Temperature Measurement in Plasmonic Nanoapertures Used for Optical Trapping. *ACS Photonics* **2019**, *6* (7), 1763–1773. https://doi.org/10.1021/acsphotonics.9b00519.

(10) Baffou, G.; Girard, C.; Quidant, R. Mapping Heat Origin in Plasmonic Structures. *Phys Rev Lett* **2010**, *104* (13). https://doi.org/10.1103/PhysRevLett.104.136805.

(11) Proulx-Curry, P. M.; Chasteen, N. D. *COHDIHAflOH CHEHISTR Y REVIEWS Molecular Aspects of Iron Uptake and Storage in Ferritin*; 1995; Vol. 144. https://doi.org/10.1016/0010-8545(95)01148-I.

(12) Aier, I.; Varadwaj, P. K.; Raj, U. Structural Insights into Conformational Stability of Both Wild-Type and Mutant EZH2 Receptor. *Sci Rep* **2016**, *6*. https://doi.org/10.1038/srep34984.
